# Combined Application of Citric Acid and Cr Resistant Microbes Improved Castor Bean Growth and Photosynthesis while It Alleviated Cr Toxicity by Reducing Cr^+6^ to Cr^3+^

**DOI:** 10.3390/microorganisms9122499

**Published:** 2021-12-02

**Authors:** Shafaqat Ali, Muhammad Waseem, Afzal Hussain, Muhammad Rizwan, Awais Ahmad, Naeem Khan

**Affiliations:** 1 Department of Environmental Sciences and Engineering, Government College University, Faisalabad 38000, Pakistan; mrazi1532@yahoo.com; 2 Department of Biological Sciences and Technology, China Medical University, Taichung 40402, Taiwan; 3 Department of Microbiology, Government College University, Faisalabad 38000, Pakistan; muhammad.waseem@gcuf.edu.pk; 4 Department of Environmental Sciences, The University of Lahore, Lahore 54000, Pakistan; afzaalh345@gmail.com; 5 Departamento de Quimica Organica, Universidad de Cordoba, 14014 Cordoba, Spain; awaisahmed@gcuf.edu.pk; 6 Department of Agronomy, Institute of Food and Agricultural Sciences, University of Florida, Gainesville, FL 32611, USA

**Keywords:** chromium, citric acid, microbes, castor bean, antioxidant enzyme, phytoextraction

## Abstract

Chromium is highly harmful to plants because of its detrimental effects on the availability of vital nutrients and secondary metabolites required for proper plant growth and development. A hydroponic experiment was carried out to analyze the effect of citric acid on castor bean plants under chromium stress. Furthermore, the role of two chromium-resistant microorganisms, *Bacillus subtilis* and *Staphylococcus aureus*, in reducing Cr toxicity was investigated. Different amounts of chromium (0 µM, 100 µM, 200 µM) and citric acid (0 mM, 2.5 mM, and 5 mM) were used both alone and in combination to analyze the remediation potential. Results showed that elevated amounts of chromium (specifically 200 µM) minimized the growth and biomass because the high concentration of Cr induced the oxidative markers. Exogenous citric acid treatment boosted plant growth and development by improving photosynthesis via enzymes such as superoxide dismutase, guaiacol peroxidase, catalase, and ascorbate peroxidase, which decreased Cr toxicity. The application of citric acid helped the plants to produce a high concentration of antioxidants which countered the oxidants produced due to chromium stress. It revealed that castor bean plants treated with citric acid could offset the stress injuries by decreasing the H_2_O_2_, electrolyte leakage, and malondialdehyde levels. The inoculation of plants with bacteria further boosted the plant growth parameters by improving photosynthesis and reducing the chromium-induced toxicity in the plants. The findings demonstrated that the combination of citric acid and metal-resistant bacteria could be a valuable technique for heavy metal remediation and mediating the adverse effects of metal toxicity on plants.

## 1. Introduction

Chromium is essential in the industry because it is used in wood preservation, textile dyeing, chromium plating, and tanning. However, the uncontrolled discharge of chromium from industries makes it a prominent contaminant in both developed and developing countries such as Pakistan [1]. The discharge of chromium in high concentrations from industries has a detrimental effect on human health globally. The aqueous medium contains chromium in the form of hexavalent chromium (Cr VI) or trivalent Cr(III) [2].

In contrast to trivalent chromium (Cr^+3^), most toxicity is found in chromate (Cr^+6^), which is added to the environment from industrial waste [1]. It has very efficient transportability from the membrane as a solid power to be oxidized [3]. The eukaryotes and prokaryotes can transport the chromate across the cell membranes actively [4]. It is found that there is the involvement of chromate transport across the membranes of biological systems byways of up-taking sulfate [5]. This phenomenon is observed in the membranes of bacteria such as *Escherichia coli, Salmonella typhimurium, Alcaligenes eutrophus,* and *Pseudomonas fluorescens* [6]. Because chromate contains an oxyanion, the anionic components of the cell membrane are unable to trap it. When cells face chromium entrance, diseases such as cancer, asthma, allergic reactions, organ failure, cardiovascular and nervous system disorders may develop [7]. Wastewater treatment industries worldwide use various physicochemical processes to handle chromium-polluted wastewater, such as filtration, ion exchange, electrochemical application, chemical precipitation, evaporation, reduction, reverse osmosis, and oxidation [8,9,10]. However, less than 8% of wastewater is treated effectively before discharge in surface water bodies in Pakistan and many other developing countries by using these techniques [11]. However, these techniques are also responsible for the pollution of the environment due to their harmful byproducts [12]. On the other hand, bioremediation is gaining popularity as a green and cost-effective alternative [13].

*Ricinus communis* L. (castor bean) is a fast-growing perennial crop cultivated in semiarid, subtropical agro-climatic conditions. It is widely grown in the subcontinent’s arid region and has tremendous ability to grow on degraded and saline soils [14]. Castor beans have gained considerable importance in remediating metals-contaminated soil due to their ability to uptake and tolerate high concentrations of heavy metals such as Cu, Fe, Mn, Zn, Cd, and Pb [15]. Castor bean is also an essential source of non-food oil for biofuel production and use in medicine and cosmetics. Although castor bean has a natural ability to withstand toxic metal concentrations, a high concentration of metals has a detrimental effect on plant growth and development, as reported by many researchers [16]. 

Various synthetic and natural chelators are being used to increase the bioavailability of metals in contaminated media [17]. Among synthetic chelating agents, ethylenediaminetetraacetic acid (EDTA) and diethylenetriamine penta acetic acid (DTPA) are widely used because they are effective at complexing metals and increasing their concentration in plant upper parts [18]. Most synthetic chelators are non-biodegradable and can cause soil contamination in the long run [19]. In contrast, organic chelating agents are low-molecular-weight organic acids such as citric acid (CA) that can form complexes with heavy metals and have a higher degree of biodegradability and less leaching hazard than synthetic chelating agents. Many studies have reported the prominent role of CA and other organic chelating agents on the extraction of heavy metals from contaminated soils and solutions cultures [20]. The CA has also been reported to improve plant growth and development under toxic metal stress [21]. 

**Figure 1:**
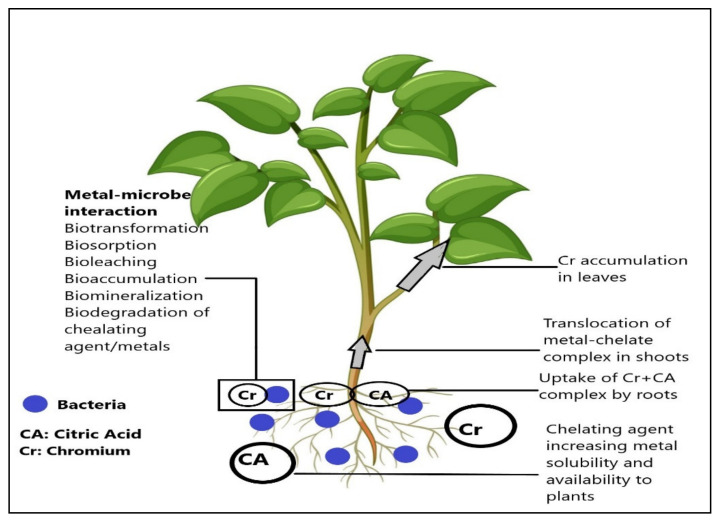
Graphical representation of a chelating agent, bacteria, and heavy metal removal process.

*Bacillus subtilis* and *Staphylococcus aureus* are a potential, gram-positive module bacteria mainly used for cellular metabolism, genetics, and resistance against heavy metals [22,23]. These bacteria have been used as a cell factory to manufacture enzymes, functional carbohydrates, and vitamins. These bacteria are also used for nutraceutical production because they are generally considered safe (GRAS) [24]. In phytoremediation, these bacteria have tremendous potential because of resistance against heavy metals such as Pd, Zn, Ni, Cr, and Co [25,26]. The interaction between the chelating agent, bacteria, and heavy metals is presented in Figure 1.

The use of metal-resistant bacteria is one of the environmentally friendly and cost-effective techniques for wastewater treatment. Bacteria develop constant contact with the pollutants in the polluted water and adapt to these harsh and unfavorable conditions. [27]. The industrial effluent contaminated with metals can be remediated by using these bacteria [28]. This study aimed to investigate the bacteria in industrial effluent to use in environmental remediation techniques. Previously, little research was conducted on a single type of Cr-reducing microorganism to alleviate metal stress. However, to the best of our knowledge, this is the first study to look at the combined effect of two Cr-reducing strains and citric acid application for Cr phytoremediation in castor bean plants.

## 2. Materials and Methods

### 2.1. Isolation of Chromium (Cr) Resistant Bacteria

The isolation of metal tolerant bacteria from the metal-contaminated wastewater samples was done by utilizing the modified serial dilution method [29]. Distilled water was used to make the final volume of the dilution solutions of wastewater samples up to 100 microliters. All dilution samples were poured onto Petri plates. These Petri plates contained 20 mL tryptic soy agar incorporated with 0.5 mM hexavalent (Cr^6+^). Those colonies were purified on Petri plates that were morphologically different. Afterward, these colonies were tested with elevated Cr concentrations to tolerate their ability by culturing them on Petri dishes. With increasing concentrations, i.e., 0.0 mM, 0.5 mM, 2.0 mM, 5.0 mM, 10.0 mM, 15.0 mM, 18.0 mM, 20.0 mM, 22.0 mM, and 23.0 mM of hexavalent chromium (Cr^6+^), these Petri dishes were augmented [30]. For further studies, only chromium-resistant bacteria were selected [31].

### 2.2. Bacterial Identification

The extraction of genomic DNA was performed by “Favorgen^®^ genomic DNA extraction kit” to characterize molecules. By utilizing universal primers set such as by polymerase chain reaction “1492R (5′-ACGGGCGGTGTGTAC-3′)” and “27F (5′-AAACTCAAATGAATTGACGG-3′)”, the gene of 16S rDNA was augmented [32]. Initially, the denaturing temperature of 94 °C was employed for 5 min of PCR. Then, the temperature of 94 °C was used to denature the 40 recurring cycles for 30s and then a temperature of 53 °C was used for annealing. In the end, the thirty-second duration of elongation was performed at the temperature of 72 °C.

Following the temperature at 4 °C, the final extension time was 10 min at 72 °C. Under UV light, by utilizing the Gel Documentation System, the amplicon of the polymerase chain reaction was demonstrated by loading the 5 μL product of polymerase chain reaction on the gel of agarose [33]. For sequencing, 25 μL of the polymerase chain reaction product was relayed to Macrogen (Seoul, Korea) after confirmation. By utilizing the software of “ChormasPro (v1.7.1)”, the nucleotide sequence was corrected manually. After this, it was submitted for accession number to GenBank. Similar sequences were observed, searched, and downloaded for the phylogenetic tree construction through the blast analysis of “NCBI” by utilizing partial sequences gene of 16S rDNA through “MEGA (v7.0.)” computer software [34].

### 2.3. Microbial Inoculum Preparation

Under sterilized conditions, the isolated bacteria obtained from tannery effluent were refreshed using a nutrient agar medium. To manufacture heavy loads of Cr reducing microbes, these colonies were cultured into a nutrient broth medium. Then, these flasks were placed in a shaking incubator with a speed of 200 rpm for 48 h at 30 °C. At the speed of 6000 rpm, broth media containing microbial culture was centrifuged for 10 min after 2 days (Beckman Co., Lakeview Parkway S Drive Indianapolis, IN 46268, USA). These microbial pellet cells were resuspended by utilizing distilled water and, concerning cell density of 108 CFU mL^−1^, adjusted the optical density at OD 660 = 0.08 [35].

### 2.4. Hydroponic Experiment

Castor bean (DS-30 cultivar) seeds were washed with distilled water and sown in sand media. After 3 weeks of seeding, healthy seedlings were transplanted into a 50 L container containing Hoagland nutrient solution. The half-strength nutrition solution was applied first, followed by the full-strength nutrient solution. The nutrient solution was changed every six days, and an electric pump was installed to provide continuous aeration. After 15 days, different amounts of hexavalent Cr (0, 100, 200 M) were added to the nutritional solution using K_2_Cr_2_O_7_. Following one week of Cr treatments, different levels of CA (0, 2.5, and 5 mM) were applied in a complete randomized design.

### 2.5. Plants Harvesting

When the castor bean plants reached maturity, they were harvested, and their various parts were separated. After washing with distilled water, plants were dried at 70 °C for 72 h, and their dry weights were recorded. The washing of roots was done by diluted hydrochloric acid (1.0%), and then distilled H_2_ O was used to wash them several times to remove the acid altogether. These roots were first dried at room temperature and then oven dried at 70 °C; the dry weight was noted. 

### 2.6. Chlorophyll Contents and Gas Exchange Parameters

The contents of chlorophyll were determined by taking fresh samples of leaves. These samples were extracted using acetone 85% (*v/v*). Centrifugation was used to estimate chlorophyll content, and a spectrophotometer was used to record readings at various wavelengths [36]. Using IRGA (infrared gas analyzer), the conductance of stomata, rate of photosynthesis, the efficacy of water use, and rate of transpiration were measured in the full sunlight of that day.

### 2.7. Estimation of MDA, EL, H_2_O_2_, and Antioxidants Enzymes

The contents of malondialdehyde were measured by following the method of Zhang and Kirkhamin and Abbas et al. by using thiobarbituric acid (0.1%) [37,38]. Electrolytes leakage (EL) was estimated by the method of Dionisio-Sese and Tobita [39]. In this regard, the solution’s EC was initially recorded by extracting the samples at 32 °C for 2 h and then extracting the same samples for 20 min at 121 °C. The contents of hydrogen peroxide were measured by following the procedure of Jana and Choudhuri [40]. The sample was homogenized (50 mM) with a phosphate buffer at a pH of 6.5 and then centrifuged for 20 min. After this sulfuric acid (20%, *v/v*) was added, it was centrifuged for another 15 min and the absorbance was measured at 410 nm.

The SOD and POD activity was measured by crushing the samples in liquid N_2_ medium and standardized in a 0.5M phosphate buffer at pH 7.8. The CAT and APX analysis was done according to Aebi [41], and Nakano and Asada [42], respectively.

### 2.8. Estimation of Cr Contents

The hot plate method was applied to digest every sample (1 g) by using HNO_3_ and HClO_4_ (*v*/*v*) in the ratio of 4:1, respectively. An atomic absorption spectrophotometer measured the total Cr concentration in these digested samples (novA A400, Analytik Jena, Jena, Germany). The Cr^+6^ concentration was determined by the diphenyl carbazide method [43].

## 3. Results

### 3.1. Plants Growth and Biomass

**Figure 2:**
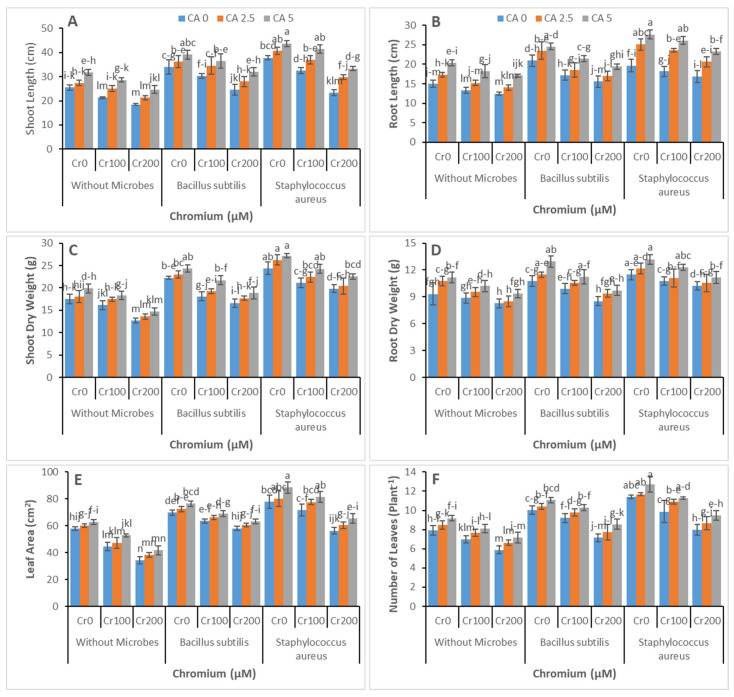
Effect of combined application of CA (0 mM, 2.5 mM, and 5 mM) and two microbes (*Bacillus subtilis* and *Staphylococcus aureus*) under Cr stress (0 mM, 100 mM, and 200 µM) on shoot length (**A**), root length (**B**), shoot dry weight (**C**), root dry weight (**D**), leaf area (**E**), and number of leaves per plant (**F**) of castor bean plants grown hydroponically. Values reported in the figures are means of 3 replicates along with standard deviation. Different lower case letters indicate significant differences among different treatments at *p* ≤ 0.05.

The interactive effect of both CA and two Cr-reducing bacterial strains on growth and Cr remediating potential of castor bean plants was observed under hydroponic conditions. The effect of CA and Cr-reducing strains on different parameters related to plant growth is shown in Figure 2A–F. Results showed decreased plant growth with increased Cr concentration, and maximum reduction was noted at Cr (200 µM). However, the application of CA and Cr-reducing strains improved the health of affected plants. Increasing the concentration of CA increased the plants’ biomass in a dose-additive manner. Even both Cr-reducing strains assisted the castor bean for Cr phytoremediation. However, *Staphylococcus aureus* showed higher potential with this regard compared with *Bacillus subtilis*. The maximum increase in castor bean growth and biomass was recorded with *Staphylococcus aureus* inoculum along with CA (5 mM).

### 3.2. Chlorophyll Contents and Gas Exchange Parameters

**Figure 3:**
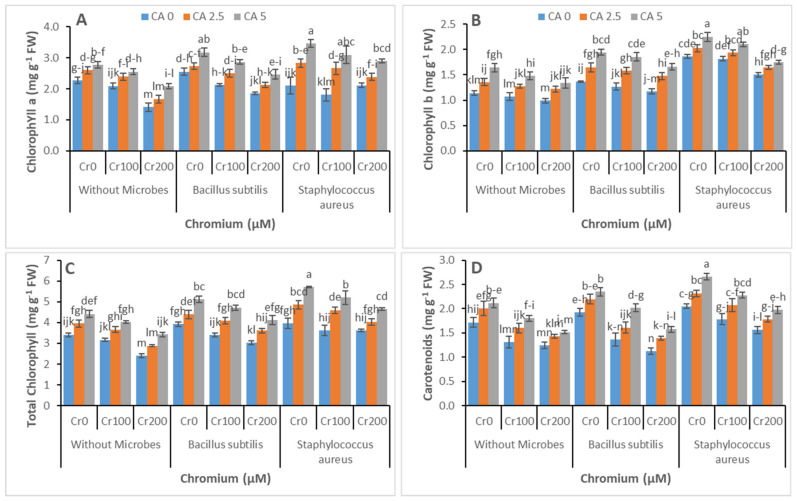
Effect of combined application of CA (0 mM, 2.5 mM, and 5 mM) and two microbes (*Bacillus subtilis* and *Staphylococcus aureus*) under Cr stress (0 mM, 100 mM, and 200 µM) on chlorophyll *a* (**A**), chlorophyll *b* (**B**), total chlorophyll (**C**), and carotenoids (**D**) of castor bean plants grown hydroponically. Values reported in the figures are means of 3 replicates along with standard deviation. Different lower case letters indicate significant differences among different treatments at *p* ≤ 0.05.

**Figure 4:**
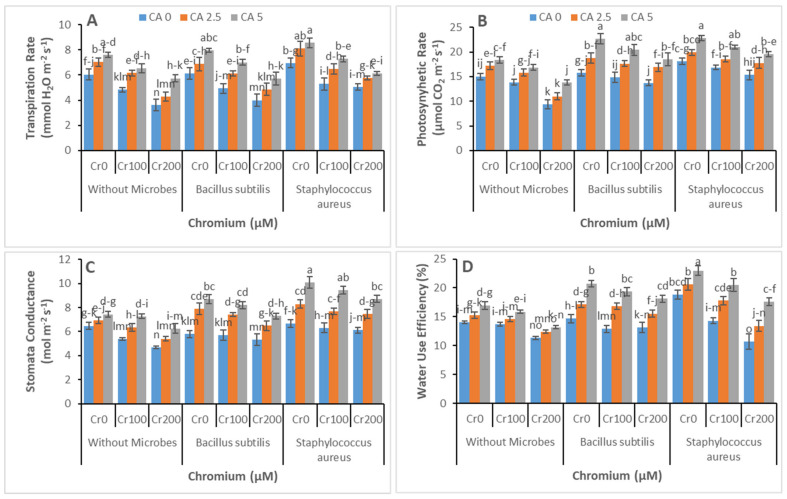
Effect of combined application of CA (0 mM, 2.5 mM, and 5 mM) and two microbes (*Bacillus subtilis* and *Staphylococcus aureus*) under Cr stress (0 mM, 100 mM, and 200 µM) on transpiration rate (**A**), photosynthetic rate (**B**), stomata conductance (**C**), and water use efficiency (**D**) of castor bean plants grown hydroponically. Values reported in the figures are means of 3 replicates along with standard deviation. Different lower case letters indicate significant differences among different treatments at *p* ≤ 0.05.

Citric acid and Cr-reducing strains significantly affected the chlorophyll and carotenoid contents and gas exchange parameters of castor bean under Cr toxicity, as shown in Figure 3A–D and Figure 4A–D, respectively. Chromium significantly reduced the chlorophyll contents and carotenoid contents along with IRGA parameters. In another way, Cr-reducing strains and CA application enhanced chlorophyll contents and gas exchange parameters. The highest values for chlorophyll contents and gas exchange parameters were observed at the highest level of CA (5 mM) and with *Staphylococcus aureus* inoculum application.

### 3.3. Electrolyte Leakage, MDA, and H_2_O_2_ Contents

**Figure 5:**
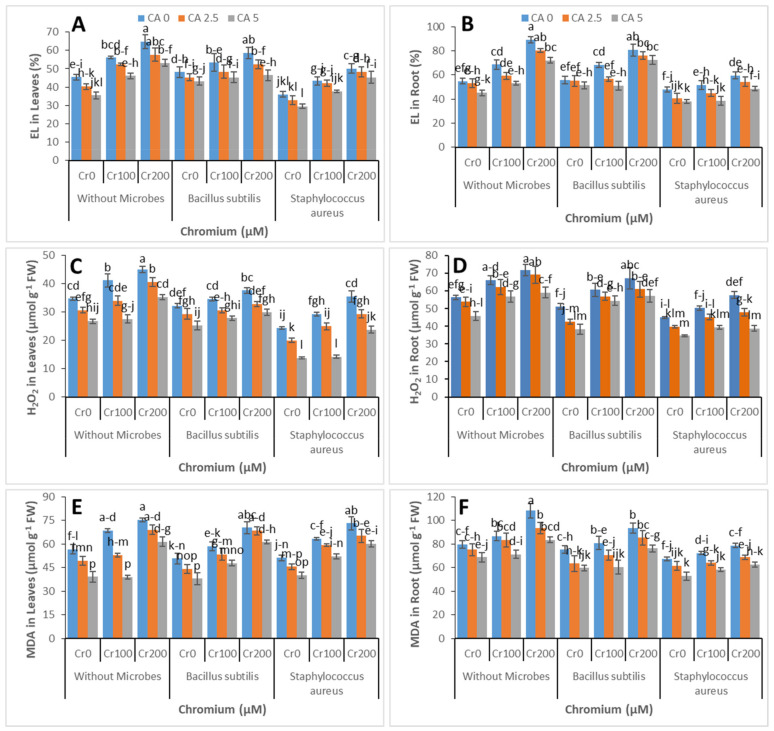
Effect of combined application of CA (0 mM, 2.5 mM, and 5 mM) and two microbes (*Bacillus subtilis* and *Staphylococcus aureus*) under Cr stress (0 mM, 100 mM, nd 200 µM) on EL in leaves (**A**), EL in the root (**B**), H_2_O_2_ in leaves (**C**), H_2_O_2_ in the root (**D**), MDA in leaves (**E**), and MDA in the root (**F**) of castor bean plants grown hydroponically. Values reported in the figures are means of 3 replicates along with standard deviation. Different lower case letters indicate significant differences among different treatments at *p* ≤ 0.05.

The effect of CA and Cr-reducing strains on the EL, MDA, and H_2_O_2_ contents of castor beans was estimated for oxidative stress observation, and the results are shown in Figure 5A–F. An increase in these parameters contents was noticed with the addition of Cr in a dose-additive manner. However, the application of CA and Cr-reducing strains reduced the oxidative damage in castor bean roots and leaves. At application of CA (5 mM) and *Staphylococcus aureus, the* maximum reduction in contents of EL, MDA, and H_2_O_2_ was recorded.

### 3.4. Antioxidants Enzymatic Activities

**Figure 6:**
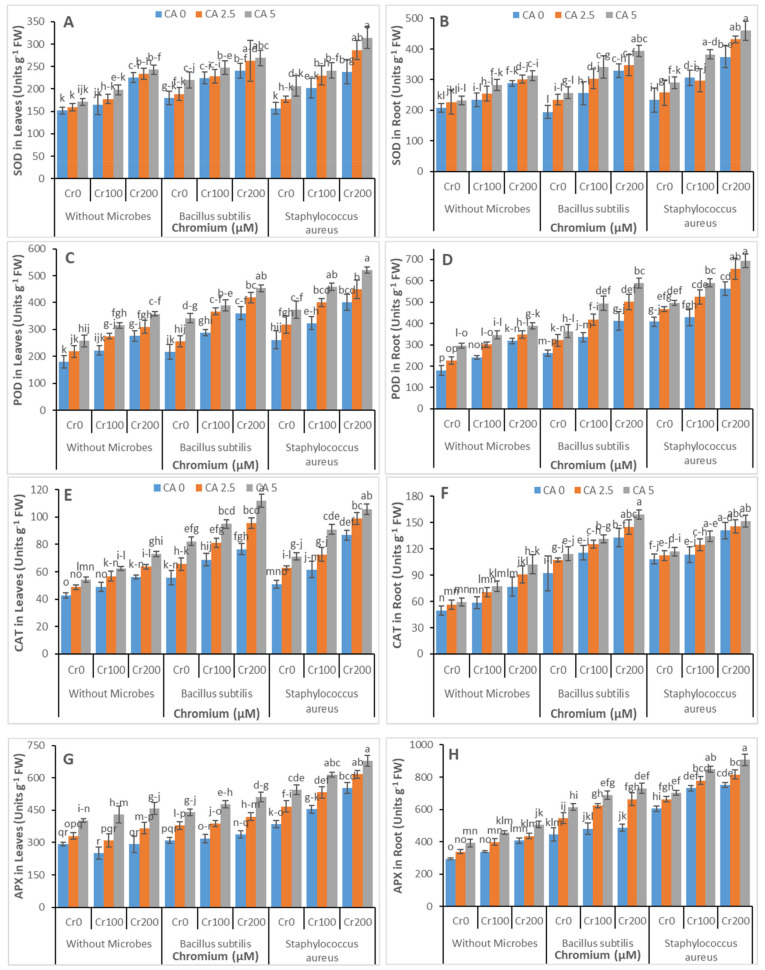
Effect of combined application of CA (0 mM, 2.5 mM, and 5mM) and two microbes (*Bacillus subtilis* and *Staphylococcus aureus*) under Cr stress (0 mM, 100 mM, and 200 µM) on SOD in leaves (**A**), SOD in the root (**B**), POD in leaves (**C**), POD in the root (**D**), CAT in leaves (**E**), CAT in the root (**F**), APX in leaves (**G**), and APX in root (**H**) of castor bean plants grown hydroponically. Values reported in the figures are means of 3 replicates along with standard deviation. Different lower case letters indicate significant differences among different treatments at *p* ≤ 0.05.

To see the enzymatic antioxidants activities, the contents for SOD, POD, CAT, and APX were estimated in leaves and roots of castor bean, represented in Figure 6A–H. These parameters were significantly affected by CA and microbial strains under Cr stress. Catalase and APX activities in castor bean leaves were significantly increased with the increasing concentration of CA. However, the maximum increase was observed at the maximum level of CA (5 mM). *Bacillus subtilis* inoculum enhanced the contents of SOD, POD, APX, and CAT compared with the control. However, the maximum increase was observed with the application of *Staphylococcus aureus* inoculum.

### 3.5. Chromium Concentration

**Figure 7:**
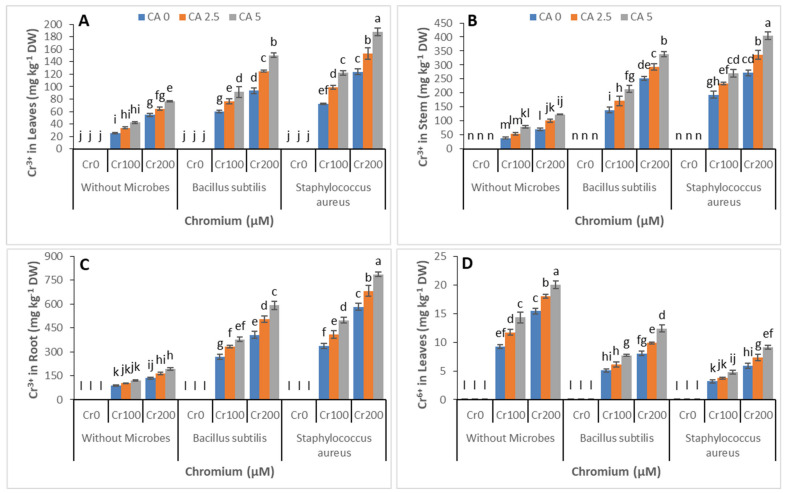

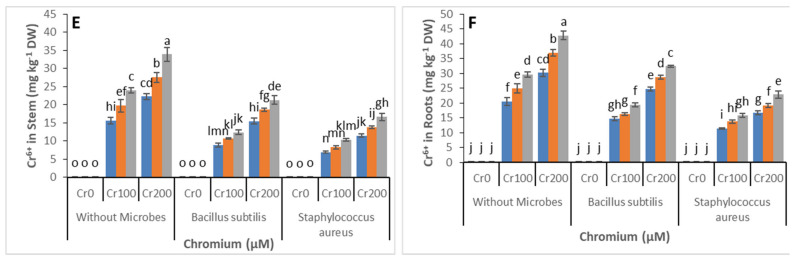
Effect of combined application of CA (0 mM, 2.5 mM, and 5mM) and two microbes (*Bacillus subtilis* and *Staphylococcus aureus*) under Cr stress (0 mM, 100 mM, and 200 µM) on Cr^3+^ in leaves (**A**), Cr^3+^ in stem (**B**), Cr^3+^ in the root (**C**), Cr^6+^ in leaves (**D**), Cr^6+^ in stem (**E**), Cr^6+^ in the root (**F**) of castor bean plants grown hydroponically. Values reported in the figures are means of 3 replicates along with standard deviation. Different lower case letters indicate significant differences among different treatments at *p* ≤ 0.05.

The effect of CA and microbial strains was observed on uptake of Cr^3+,^ and Cr^6+^ leaves, stem, and roots of castor bean under Cr stress, grown hydroponically, and results are presented in Figure 7A–F The K_2_Cr_2_O_7_ was used as a source of Cr^6+^ in nutrient solution in this experiment. We observed the uptake of both Cr^3+^ and Cr^6+^ in different parts of plants. The uptake of Cr^3+^ was increased with the addition of microbial strains. Maximum uptake of Cr^3+^ was recorded where we inoculated *Staphylococcus aureus* strains, and minimum uptake of Cr^3+^ was observed where no microbial strains were applied. The uptake of Cr^6+^ was decreased with the addition of microbial strains. Maximum Cr^6+^ uptake was observed without application of microbial strains, and minimum C^6+^ was uptaken with *Staphylococcus aureus.* The application of CA further enhanced the Cr uptake. The uptake of both Cr^3+^ and Cr^6+^ was greater in plant roots as compared with aerial parts.

The Cr accumulation was also observed in castor bean plants’ shoot and root, as shown in Figure 7A–F. Our results indicate that the application of both microbial strains facilitated the accumulation of Cr. However, the highest accumulation was recorded with *Staphylococcus aureus* at Cr (200 µM). Higher Cr accumulation was observed in castor bean shoots as compared with the root. The addition of CA enhanced Cr accumulation even more, and it was at its peak at the highest level of CA (5 mM).

## 4. Discussion

The role of citric acid and bacteria in mitigating the toxicity of Cr in castor bean seedlings is demonstrated in this study. In tannery wastewater, various heavy metals are found in trace amounts, but chromium is found in the highest concentration [44]. Metal tolerant bacteria can be isolated from sites polluted with industrial wastewater [45,46]. This study has characterized and isolated indigenous *Staphylococcus aureus* strain “K1” and *Bacillus subtilis* from the tannery effluent capable of tolerating Cr^6+^ up to 22 mM. 

When the chromium concentration in tannery wastewater was raised, different plant growth parameters, such as leaf number, biomass, plant height, leaf area, and root length, decreased (Figure 2). The increasing chromium levels in wastewater might be the reason for this decrease in growth parameters [47]. Various studies have identified a decline in the morphological features of various plant species under high concentrations of metals [48,49]. There was much evidence regarding the change in morphology induced by chromium because of chromium competition with other vital elements, induced oxidative damage, disturbed photosynthesis, and leaf and root ultrastructure distortion [50,51]. Previous studies have found chromium stress reduced plant height, biomass, and root length in various plant species such as spinach, maize, wheat, and tobacco [52,53]. The inoculation of plants with bacteria and application of CA improved the plants’ growth parameters (Figure 2). Previous studies report that bacterial inoculation may increase the nutritional requirements of micronutrients and macronutrients by altering host physiology and changing the root uptake system. In a recent study, bacterial inoculations increased Fe and K content in maize plants under Cr stress [54].

The growth and biomass of the plants of castor bean stressed by chromium were improved by applying citric acid compared with treatments treated with tannery wastewater without the exogenous application of citric acid (Figure 2). The increased necessary nutrient uptake by the castor bean and minimized chromium uptake may improve the plant features of morphology [50]. Similarly, the toxic metal effects on bacterial growth could be reduced by utilizing the rich medium of nutrients for the growth of bacteria [55]. For instance, researchers estimated the effects of several metal concentrations by utilizing the rich culture medium of nutrients composed on the extraction of yeast and tryptone on the growth of bacteria [56,57]. Chromium formed a complex structure with organic compounds under such conditions. As a result, the toxic effects of chromium were reduced, and it became less effective against microbial metabolism [58,59].

The photosynthesis pigments play a crucial role in the life of plants as they have the functionality to harvest light. The toxicity of chromium influenced the physiochemical features of plants [60]. In this work, the stress of Cr enriched wastewater decreased photosynthesis pigments significantly (Figure 3). When the amount of tannery wastewater increased, there was abrupt minimization in the contents of total chlorophyll, chlorophyll a, and chlorophyll b. The presence of chromium in tannery wastewater might be the reason behind this decrease in the plant growth characteristics, as reported by other researchers [61,62]. The chloroplast might have acquired impairment in structure [63], and the ROS formation increased under stress induced by high concentration of chromium [64]. Chlorophyll pigments were drastically reduced when chlorophyllase was activated in response to heavy metal stress [62]. The contents of chlorophyll were increased effectively when citric acid was applied to plants under chromium stress. Mallhi et al., 2020, also reported the improvement in the chlorophyll contents of the sunflower plant under the stress of chromium which was mediated by CA [20].

Similarly, chlorophyll contents were increased in maize plants subjected to drought stress and treated with citric acid [65]. The photosynthetic pigments might have been protected by the treatment with citric acid that minimized chromium uptake and minimized its translocation to the upper parts of the plant. Further, the chlorophyll contents also increased with the bacterial inoculation of plants under chromium stress. The increase in chlorophyll contents by application of bacteria may be attributed to the reduction in the availability of chromium, increase in uptake of nutrients by plants, and increased ability of plants to withstand chromium toxicity. Similar improvement was observed in chlorophyll contents of wheat plants under chromium stress treated with *Staphylococcus aureus* [66].

Citric acid successfully inhibited chloroplast damage and ROS generation (Figure 4), allowing plants to be improved with pigment content [62]. Additionally, the damage to the membrane and peroxidation of lipids was indicated by the enhanced contents of MDA [67]. In this study, there was an effective increase in the contents of MDA and hydrogen peroxide and electrolyte leakage in the leaves and roots of castor bean under Cr stress (Figure 5). However, the damage of membrane and peroxidation of lipids was amended by treating citric acid by foraging free radicals and minimizing ROS formation [68,69]. Under Cr stress, bacterial inoculation reduced lipid peroxidation, which could be related to increased ROS-scavenging enzyme synthesis in plants. Previous research has demonstrated that bacterial inoculation activated the gene profile of metal detoxifying enzymes in response to metal stress [70].

From oxidative stress, an excellent role was performed by antioxidant enzymes in defending the plants. The existing literature shows that when stress level was changed from mild to moderate, POD, APX, SOD, and CAT activities increased. The activities of SOD and POD drastically decreased on increasing the dose of wastewater, except POD in leaves. It was enhanced in leaves at higher stress levels (Figure 6); Meng et al., 2009, examined similar results [71]. It was previously investigated that the activities of antioxidant enzymes decreased at severe stress levels in maize because of the constant oxidative damage [61]. This was because chromium stress alerts the antioxidant plant machinery to begin ROS scavenging as soon as possible. However, because of enhanced and continuous ROS production, greater levels of chromium suppressed the antioxidant system. Increased antioxidant enzyme activity in bacteria-inoculated plants may be attributable to increased antioxidant enzyme mRNA/gene expression relative to uninoculated plants [67].

In the stem, roots, and leaves of castor bean plants, enhancing the Cr concentration enhanced the Cr uptake by castor bean plants (Figure 7). When roots were contrasted with the stem and leaves of the castor bean plants, roots accumulated a large amount of chromium. Similarly, the same results were reported by other researchers for rice and rapeseeds [72,73]. The sucrose as macromolecules bounded the chromium and confined it in the vacuoles of the root cell compartmentalization [60]. Under different levels of treatment of tannery wastewater, the chromium accumulation and uptake in several plant parts were diminished significantly by exogenous uses of citric acid (Figure 6). Similar results were reported by other studies in which the utilization of citric acid reduced the uptake of several metals and the upper part of the plant, their translocation occurs [20,74]. Because of the fortification of the plant membrane system and the resultant enhancement in the nutrient uptakes, the negotiated citric acid decreased chromium absorption, increasing the plant’s growth [74]. For their constructive effects, organic substances, for example, humic acid and fulvic acid, are well known for the bioavailability and mobility of heavy metals [75]. In this way, they form the complexes of organo–metals, as citric acid made complexes with chromium ions which might be the reason for the decrease in chromium uptake [76,77].

As chromium belongs to the d block series in the periodic table, known as transition metals, it has different transition states. It exists as Cr^6+^ and Cr^3+^. Due to this, Cr has a vast range of properties in terms of bioavailability and mobility. The Cr^6+^ was known as more virulent rather than Cr^3+^ [78]. In this study, there has been an increase in leaf and root Cr contents mediated with the addition of microbes in Cr-media. Due to the acidic conduct of citric acid oxidation, the state of Cr changes from hexavalent to trivalent, which is less toxic and uptaken by the castor bean plant without detrimental effect on plant growth and development [79,80]. In our study, 2.5 mM of citric acid amendment of Cr-media resulted in a significant accumulation of Cr^3+^ in aerial parts compared with control plants which exhibited more endogenous levels of Cr^6+^.

The absorption behavior of Cr media in plants was reduced by adding 5 mM of Cr media with citric acid. Microbes have a potential effect for the protection of plants from the phytotoxic effect of Cr^6+^ by converting its oxidation state to Cr^3+^, which is less toxic. However, we recorded a drastic decline in growth characteristics of plants with the 5 mM EDTA amendment of Cr media. It could be related to an increase in endogenous Cr^3+^ contents, as reported by the previous study [66]. It is well known that Cr^3+^ becomes toxic for plant growth and biomass production when accumulated in higher concentrations [52]. The toxic metals can slow bacterial growth; similarly, in this study, the slower growth rate of bacteria was due to the high concentration of chromium, i.e., 22 mM [81,82,83]. Our result correlated with previous studies as they reported growth reduction in spinach and sunflower plants grown under high concentrations of chromium [44,79]. Both bacterial strains effectively contributed to remediating the toxicity induced by chromium in castor bean plants. However, the plants’ growth, photosynthetic parameters, and antioxidant enzymes production were significantly better in plants inoculated with *S. aureus* than *B. subtilis*. It might be attributed to the better ability of *S. aureus* to tolerate and remediate high concentrations of chromium over *B. subtilis* [66,84]. 

## 5. Conclusions

In contrast to remediation of chromium, the microbes such as *Bacillus subtilis* and *Staphylococcus aureus* demonstrate the auspicious effect for stress behavior. However, the capacity of the castor bean has been limited due to the phytotoxic effect of metal phytoremediation. It has been observed that the growth of the castor bean plant minimizes under chromium stress. In plants, their effect is minimized as phytotoxic because of the oxidative behavior of Cr that exists in two different oxidation states, Cr^3+^ instead of Cr^6+^. These two microbes, *B. subtilis* and *S. aureus*, have excellent potential to tolerate chromium toxicity and remediate chromium from the polluted medium. Furthermore, the application of CA under high chromium concentrations can enhance the capacity of the plants to tolerate and remediate chromium. The chromium-contaminated soil can be remedied using the castor bean plant and microbes.
